# Response: Commentary: Zika Virus: the Latest Newcomer

**DOI:** 10.3389/fmicb.2016.01398

**Published:** 2016-09-07

**Authors:** Juan-Carlos Saiz, Ana B. Blázquez, Nereida Jiménez De Oya, Teresa Merino-Ramos, Miguel A. Martín-Acebes, Estela Escribano-Romero, Ángela Vázquez-Calvo

**Affiliations:** Department of Biotechnology, Instituto Nacional de Investigación y Tecnología Agraria y AlimentariaMadrid, Spain

**Keywords:** Zika virus (ZIKV), Guillain-Barre syndrome, Flavivirus, surveillance, communicable diseases

We really appreciate Craig et al. (2016) for their commended to our contribution to the current knowledge about Zika Virus (ZIKV), and their comments to the possible relationship between Guillain-Barre syndrome (GBS) and ZIKV infection (Saiz et al., 2016). Even more, we absolutely agree with their interpretations and recommendations pointing to the necessity of further research to accurately quantify the effect of enhanced surveillance related to ZIKV circulation and associated risks. In fact, we clearly stated in the abstract of our review (Saiz et al., 2016) and throughout the text, including our final remarks, that clarifying whether there is a causal link between ZIKV infection and GBS is a currently unavoidable goal.

In this regard, recently, we have specifically addressed what it is currently known about ZIKV infections in relation with neurological manifestations (Blázquez and Saiz, in press), and stated that, beside the reported data pointing to a relationship between ZIKV infection and GBS, so far, a causal association has not been yet solidly established.

GBS is a clinical syndrome of multiple autoimmune etiologies and the most common and severe acute paralytic neuropathy (van den Berg et al., 2014; Willison et al., 2016). In in many cases GBS appears to be associated with antecedent infectious diseases (Winner, 2001), and sporadic arboviral infections with dengue virus, DENV (Garg et al., 2015; Simon et al., 2016), West Nile virus, WNV (Sejvar, 2004), or chikungunya virus, CHIKV (Wielanek et al., 2007) have already been associated to GBS. In fact, Malone et al. (2016) have suggested that the concomitant regional increase in DENV and CHIKV infections may have contribute to the recently registered increase in GBS incidence.

In any case, and beyond the considerable efforts carried out by the scientific community and the national and international health authorities focused on deciphering ZIKV infection and its consequences, sufficient resources should be allocated to provide the necessary tools to evaluate the potential mechanisms of ZIKV association to GBS.

## Author contributions

All authors conceived the ideas presented in the article. J-CS led the drafting of the article with inputs from all other contributors. AV-C led the submission process.

## Funding

This work was supported by grant ZIKA-BIO from INIA to J-CS, and AGL2014-56518-JIN from MINECO to MM, AV-C is a recipient of a “Contrato de formación postodoctoral” from MINECO. TM is a recipient of a “Formación de Personal Investigador (FPI)” pre-doctoral fellowship from INIA.

### Conflict of interest statement

The authors declare that the research was conducted in the absence of any commercial or financial relationships that could be construed as a potential conflict of interest.
